# Impact of the COVID-19 Pandemic on Alcohol Treatment Access and Harm Prevention in West Africa: Reports from NGOs and Community-Based Organizations

**DOI:** 10.1007/s44197-022-00035-7

**Published:** 2022-04-05

**Authors:** Monica H. Swahn, Adelaide Balenger, Franklin Umenze, Ritu Aneja, Thomas A. Bureh, Emeka W. Dumbili, Isidore Obot

**Affiliations:** 1 Wellstar College of Health and Human Services, Kennesaw State University, Prillaman Hall, Room 4103, 520 Parliament Garden Way, Kennesaw, GA 30144 USA; 2 School of Public Health, Georgia State University, Atlanta, GA USA; 3 Preston Hospital Lekki, West African Alcohol Policy Alliance, Lagos, Nigeria; 4 West African Alcohol Policy Alliance, Accra, Ghana; 5 College of Arts and Sciences, Georgia State University, Atlanta, GA USA; 6 Research and Documentation Center On Human Rights, Kampala, Uganda; 7 Institute for Therapy and Health Research, Kiel, Germany; 8 Department of Sociology and Anthropology, Nnamdi Azikiwe University, Anambra State, Nigeria; 9 Centre for Research and Information On Substance Abuse (CRISA), Uyo, Nigeria

**Keywords:** Alcohol prevention, Alcohol harm, COVID-19 pandemic, West Africa

## Abstract

**Background:**

Recent research highlights how the COVID-19 pandemic has significantly impacted alcohol consumption patterns, yet research thus far has largely overlooked the experience in West Africa. Research also has not addressed how the COVID-19 pandemic has affected access to alcohol treatment, support, and alcohol harm prevention. This study addresses this research gap in West Africa, a low-resource setting with a very high burden of alcohol harm.

**Objectives:**

To understand the impact of the COVID-19 pandemic on alcohol use, access to alcohol, treatment access, and alcohol harm prevention activities in West Africa.

**Methods:**

This study analyzed data from a cross-sectional online survey conducted in August and September of 2020 and distributed by the West Africa Alcohol Policy Alliance to their member alliances and stakeholders across nine countries (*N* = 140 participants) to understand their perceptions on COVID-19 and alcohol-related topics.

**Results:**

Our findings convey a significant adverse impact on alcohol-focused NGOs and community-based organizations in West Africa. Overall, 94% of participants indicated that the COVID-19 pandemic adversely impacted their organizations’ work. In addition, 71% of participants reported reduced access to alcohol treatment or support in their communities. Lastly, 44% of the respondents indicated that people in their community drank less alcohol than usual, and only 33% answered that they perceived it to be harder to get alcohol.

**Conclusions:**

These data underscore the significant impact of the COVID-19 pandemic across West Africa with respect to accessing alcohol treatment and organizational capacity to address alcohol harm. With the lack of infrastructure to address alcohol harm, this impact could exacerbate the high level of alcohol use and harm in the region.

## Introduction

After COVID-19 was classified as a pandemic, spreading globally to nearly all corners of the world, emerging evidence suggested that alcohol use is closely intertwined with the COVID-19 pandemic and the associated mitigation steps to curb the virus’s transmission (such as lockdown policies). Researchers have analyzed how COVID-19 lockdowns have impacted alcohol consumption patterns [1–4]. Nevertheless, much of this research has focused primarily on countries in Europe, North America, and Asia. The impact of the COVID-19 pandemic on alcohol consumption in Africa remains largely unknown. In addition, research to date has not addressed the potential impact of the COVID-19 pandemic on access to alcohol treatment and alcohol harm prevention in West Africa. Given recent findings highlighting West Africa as the region within Sub-Saharan Africa with the highest number of age-standardized alcohol-attributable deaths and disability adjusted life years [5], disruption to treatment access and alcohol harm prevention in this region may have dire consequences.

As noted, previous research has examined how COVID-19 lockdown policies have changed alcohol consumption patterns. Ammar and colleagues conducted a survey with 40% of respondents from Africa (mostly North Africa), to examine changes in binge drinking before and during confinement measures for COVID-19 [6]. They found that the percentage of respondents who reported alcohol binge drinking was lower during confinement [6]. In contrast, researchers in the United States have found greater odds of increased alcohol consumption during the pandemic among specific groups, such as parents with children, individuals with moderate to severe depression, and those who experienced a primary job loss [1, 2]. In the Global Drug Survey, 43% of respondents reported an increase in the frequency of alcohol consumption during the pandemic [3]. Researchers in the United Kingdom reported that the proportion of people drinking four or more times per week increased during lockdown [4]. Notably absent in the rapidly growing alcohol and COVID-19 literature is research from West Africa and the pandemic’s impact on alcohol treatment, support services, and alcohol harm prevention.

Policies that restrict access to alcohol during the COVID-19 pandemic are an important contextual factor. Although outside of West Africa, a case study from South Africa illustrates the potential impact of alcohol-specific restriction policies. South Africa implemented three bans on alcohol sales since the beginning of the pandemic [7]. Reuter and colleagues noted the first ban’s dramatic effect on trauma visits to a rural hospital in South Africa, with significant declines in patients seen for assaults and injuries, and sexual assaults after the ban [8]. In West Africa, more general lockdown policies, not specific to just alcohol, could still have impacted access to alcohol and treatment. Researchers have compiled a country-level COVID-19 stringency index based on nine government response indicators, including school and workplace closures, travel bans, cancellation of public events, and restrictions on gatherings [9]. This composite measure can be used to generally gauge the degree of lockdown in each country. In West Africa, Nigeria had one of the highest stringency indices in the region as of August 1, 2020 (stringency index = 73.2), while Senegal had one of the lowest in the region (32.4). A more restrictive environment could limit access to alcohol, or alternatively, could induce alcohol consumption, as researchers elsewhere have noted [10]. However, there are no published studies using a systematic approach to assess the impact of these lockdown policies on access to alcohol, alcohol consumption patterns, access to treatment/support services, and alcohol harm prevention activities in West Africa.

Within this context, we conducted our survey in August and September of 2020 to understand the effect of the COVID-19 pandemic on alcohol use, access to alcohol, treatment access, and alcohol harm prevention in West Africa by engaging stakeholders primarily from non-governmental organizations (NGOs) and community-based organizations (CBOs) and across nine countries. A wave of COVID-19 infections hit West African countries over the summer of 2020, a few months after the dramatic increase of cases in the U.S. and Europe [11]. As such, our survey implementation coincided with the first significant wave of cases across West Africa.

## Methods

The research team created the cross-sectional West African Alcohol Policy Alliance Capacity Assessment Survey (WAAPACAS) to distribute to stakeholders involved in alcohol prevention, outreach, and policy development in West Africa. The main variables of this study were measured with four yes/no questions developed by the research team about COVID-19 and alcohol. Participants responded to the prompt, “In your opinion, have any of the following happened in your community because of COVID-19?” “People in  my community (1) Drink less than usual; (2) Find it harder to get alcohol; and (3) Find it harder to access alcohol treatment or support when needed”. These questions assess the respondents’ perceptions of changes in alcohol consumption, access to alcohol, and access to alcohol treatment and support in their communities, but do not directly measure changes since the respondents answer on behalf of their communities. Another question asked whether the COVID-19 pandemic has negatively impacted the respondents’ organization and their work.

Individuals working at predominantly NGOs and CBOs involved in alcohol prevention and alcohol policy development in West Africa responded to this online survey in August and September 2020, as part of a partnership with the West African Alcohol Policy Alliance (WAAPA). The survey was initially distributed to nine countries (i.e., Benin, Burkina Faso, The Gambia, Ghana, Guinea Bissau, Liberia, Nigeria, Senegal, and Sierra Leone), but individuals from twelve countries ultimately responded to the survey. WAAPA distributed the survey to its member alliances and contacts, and the first wave of respondents forwarded this survey to any additional contacts within their countries involved in alcohol harm prevention. The survey received Institutional Review Board approval at Georgia State University, and the survey was determined to be exempt (H21075). Participants consented to the survey prior to completing the questionnaire. They received invitations via email and on social media platforms (WhatsApp and Facebook) for the Qualtrics survey. Respondents did not receive any compensation for taking the survey. Overall, 140 participants responded to the survey, although some participants did not answer every question, so the sample size is less than 140 for some questions.

Both descriptive and inferential statistical analyses were computed. For the four survey questions related to COVID-19, country-level statistically significant differences (*p* < 0.05) in the proportion of respondents who answered yes to each question were tested using Fisher’s exact test, since some expected cell sizes were less than 5. Analyses were conducted in both Excel and SAS. In addition, the research team included the COVID-19 stringency index as of August 20, 2020 for each country included in this study [9].

## Results

Among the 140 respondents, 56% represent NGOs, 24% CBOs, and the remaining 20% represent governmental or international organizations, universities/research institutes, or other. In terms of populations that these organizations serve, 27% serve communities, 22% primary/secondary schools, 14% tertiary/vocational institutes, 12% policy/legislation/decision-makers, 12% local councils, 10% service providers, and 2% other. The countries, sample sizes, and corresponding COVID-19 stringency indices as of August 1, 2020 include: Sierra Leone (*n* = 33, 32.4), Nigeria (*n* = 32, 73.2), the Gambia (*n* = 17, 41.2), Liberia (*n* = 16, 64.8), Ghana (*n* = 13, 52.8), Senegal (*n* = 12, 32.4), Burkina Faso (*n* = 8, 56.5), Gabon (*n* = 2, 77.8), Guinea Bissau (*n* = 1, stringency index n/a), Zambia (*n* = 1, 50.9), Benin (*n* = 1, 41.7), and Guinea (*n* = 1, 77.8), as displayed in Table 1.

**Table 1:** Countries represented by respondents in the West African Alcohol Policy Alliance Capacity Assessment Survey (WAAPACAS)

Country	Count	%	COVID-19 Stringency Index, as of Aug. 1, 2020 [9]
Sierra Leone	33	24	32.4
Nigeria	32	23	73.2
The Gambia	17	12	41.2
Liberia	16	12	64.8
Ghana	13	9	52.8
Senegal	12	9	32.4
Burkina Faso	8	6	56.5
Gabon^a^	2	1	77.8
Guinea Bissau^a^	1	1	n/a
Zambia^a^	1	1	50.9
Benin^a^	1	1	41.7
Guinea^a^	1	1	77.8
Grand total^b^	137	100	

^a^Omitted from specific country analyses because of small sample sizes.

^b^Note that 3 did not answer this question.

**Chart 1:**
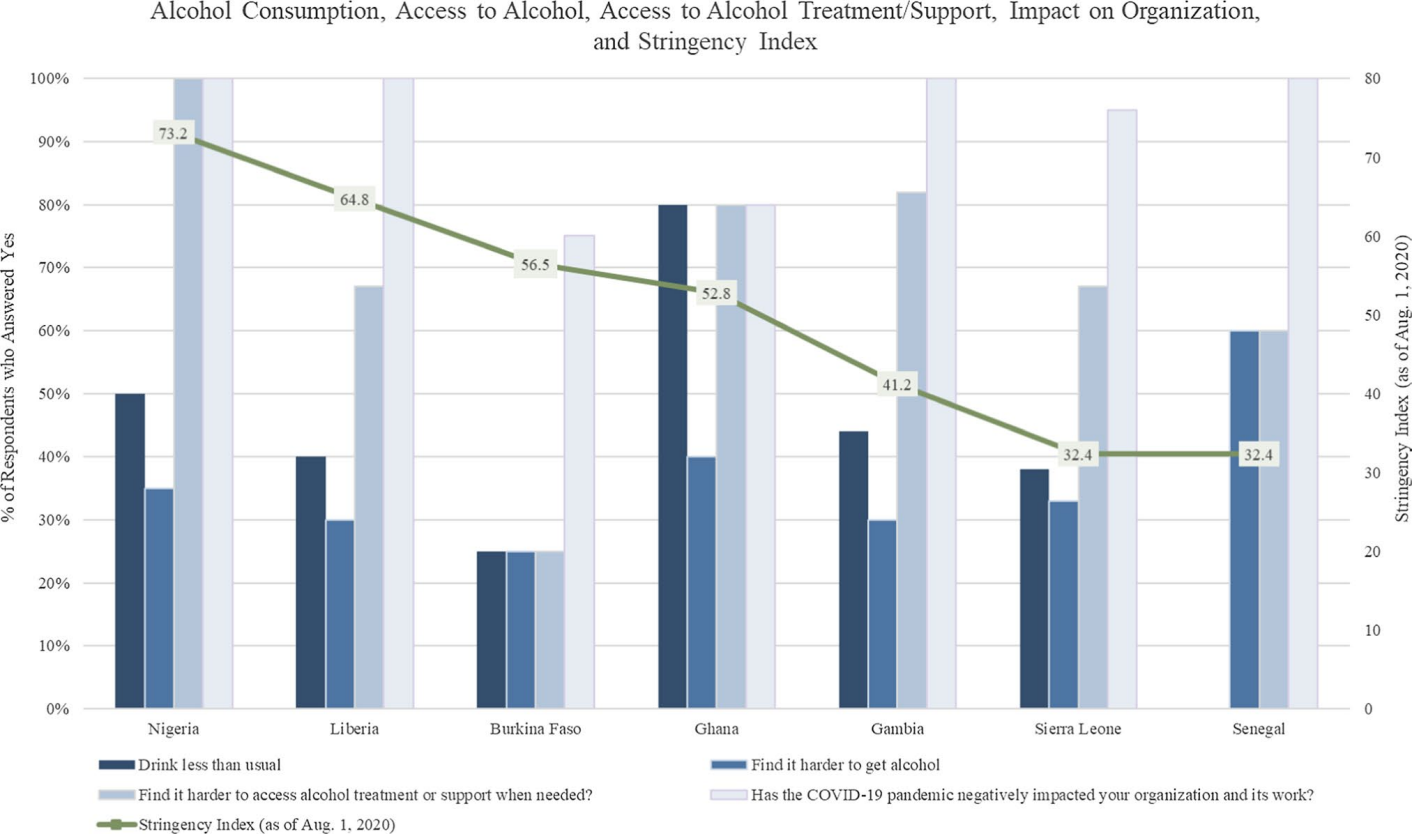
Graphical representation of COVID-19 question results from the West Africa Alcohol Policy Alliance Capacity Assessment Survey (WAAPACAS)* and the COVID-19 Stringency Index (as of Aug. 1, 2020) * Note that we included country-level results only for countries with at least five respondents, so the country-level results do not aggregate to the overall row.

Table 2 presents the overall and country-level results for the COVID-19 related questions, and Chart 1 provides a graphical representation of the country-level results for all countries with at least five respondents. The two primary findings are that the COVID-19 pandemic has made it more challenging to access alcohol treatment/support and has negatively affected alcohol prevention work. Overall, 71% of participants responded that it was harder for people in their community to access alcohol treatment or support, ranging from 25% in Burkina Faso to 100% in Nigeria. These country-level differences are statistically significant (*p*-value = 0.003), indicating a strong association between country and perceptions on access to alcohol treatment and support. Regarding the pandemic’s impact on respondents’ organizations’ work, 94% indicated that the pandemic has negatively impacted their work. The percentage of respondents reporting that the COVID-19 pandemic had negatively impacted their organizations’ work varied from 75% in Burkina Faso to 100% in Nigeria, Liberia, The Gambia, and Sierra Leone. This finding is also statistically significant (*p*-value = 0.04), indicating an association between country and the impact on the respondents’ organizations’ work.

**Table 2:** COVID-19 question results from the West Africa Alcohol Policy Alliance Capacity Assessment Survey (WAAPACAS)

Question	% Yes	Fisher Exact Test
In your opinion, have any the following happened in your community because of COVID-19? People in my community: drink less than usual—% yes (*N* = 75)	44	
Nigeria (*N* = 18)	50	*p*-value = 0.64
Sierra Leone (*N* = 16)	38	
Liberia (*N* = 10)	40	
The Gambia (*N* = 9)	44	
Burkina Faso (*N* = 8)	25	
Ghana (*N* = 5)	80	
In your opinion, have any the following happened in your community because of COVID-19? People in my community: find it harder to get alcohol % yes (*N* = 80)	33	
Nigeria (*N* = 17)	35	*p*-value = 0.93
Sierra Leone (*N* = 18)	33	
Liberia (*N* = 10)	30	
The Gambia (*N* = 10)	30	
Burkina Faso (*N* = 8)	25	
Ghana (*N* = 5)	40	
Senegal (*N* = 5)	60	
In your opinion, have any the following happened in your community because of COVID-19? People in my community: find it harder to access alcohol treatment or support when needed? % yes (*N* = 76)	71	
Nigeria (*N* = 17)	100	*p*-value = 0.003*
Sierra Leone (*N* = 15)	67	
Liberia (*N* = 9)	67	
The Gambia (*N* = 11)	82	
Burkina Faso (*N* = 8)	25	
Ghana (*N* = 5)	80	
Senegal (N = 5)	60	
Has the COVID-19 pandemic negatively impacted your organization and its work? % yes (*N* = 94)	94	
Nigeria (*N* = 22)	100	*p*-value = 0.04*
Sierra Leone (*N* = 21)	95	
Liberia (*N* = 11)	100	
The Gambia (*N* = 13)	100%	
Burkina Faso (*N* = 8)	75%	
Ghana (*N* = 5)	80%	
Senegal (*N* = 7)	100%	

*Note that we included country-level results only for countries with at least five respondents, so the country-level results do not aggregate to the overall row.

The impact of the pandemic on alcohol consumption patterns and access to alcohol also varied by country, although these differences were not statistically significant (Table 2). Overall, 44% of the respondents indicated that people in their community drank less alcohol than usual because of COVID-19, ranging from 25% in Burkina Faso to 80% in Ghana. Only 33% answered that it was harder to get alcohol because of the pandemic, with country-level percentages ranging from 25% in Burkina Faso to 60% in Senegal. Additional country-level results (those with at least five respondents) are displayed in Table 2 and Chart 1.

## Discussion

Our study, conducted in 2020 coinciding with the first wave of COVID-19 cases in West Africa, analyzed how the COVID-19 pandemic has affected alcohol use, access to alcohol, treatment access, and alcohol harm prevention based on the perceptions of individuals working in alcohol prevention-focused CBOs and NGOs across nine West African countries. Overall in the region, the majority of survey participants said that reduced access to alcohol treatment or support as a result of the COVID-19 pandemic is an important concern, although this variable was strongly associated with the respondents’ country of residence. Reduced access to alcohol treatment or support could exacerbate alcohol harm in these countries, particularly in a region with limited access to the vaccines available as of July 2021. As of July 2021, the percentage of the population fully vaccinated against COVID-19 was less than 3% for all countries represented in this survey [12, 13]. Similarly, our findings demonstrate a dramatic impact on CBOs and NGOs in West Africa due to the COVID-19 pandemic, as 94% of participants responded that COVID-19 adversely impacted their organizations’ work. In general, less than half (44%) of participants thought that people in their communities drank less than usual, and about a third of respondents perceived it to be harder to get alcohol in their communities because of the COVID-19 pandemic. Taken together, these findings underscore grave concerns  about the COVID-19 pandemic’s impact across West Africa with respect to alcohol-related harm. Given the low-resource settings combined with the lack of formal alcohol policies in West Africa [5], these findings are even more troubling.

When examining the findings by specific country, regional variations regarding the impact of the pandemic were substantial and sometimes in the opposite direction, as evidenced by Chart 1. For example, Nigeria and Burkina Faso presented two nuanced stories. Among Nigerian respondents, 50% said that people in their communities drank less than usual during the pandemic, yet 100% said it was more difficult to access alcohol treatment or support in their communities. In August 2020, Nigeria’s COVID-19 stringency index (73.2) was the highest among the West African countries included in this study. Our findings, in combination with the high stringency index, suggest that lockdown policies significantly impacted access to alcohol treatment in Nigeria. In contrast, only 25% of respondents from Burkina Faso said that people in their community drank less than usual, found it harder to get alcohol, and thought it was harder to access alcohol treatment and support. Compared to Nigeria, Burkina Faso’s stringency index was slightly lower (56.5), perhaps partially explaining the lower impact of lockdown policies on alcohol use, access to alcohol, and access to treatment/support. Gambian participants indicated that accessing alcohol treatment/support is now harder during the pandemic, with 82% responding yes, despite the country’s relatively lower COVID-19 stringency index (41.2). Of course, we use the COVID-19 stringency index as a proxy for the degree of restrictions imposed, so we do not expect respondents’ perceptions of how alcohol use and access to treatment/support to correlate perfectly with this index.

Respondents’ perceptions of changes in drinking patterns, access to alcohol, and access to treatment services may also be related to the type of organizations that respondents work at and the populations that their organizations serve. Respondents who work at NGOs or CBOs who serve only primary/secondary schools will likely have a different perspective than those whose organizations focus on community advocacy or health education. Country-level variations presumably emerged due to who responded to this survey from each country. Despite the variation and lack of precision in the country-level results, this small sample still signals that disruptions to alcohol treatment and support is a concern in the region, particularly so in countries such as Nigeria and The Gambia.

One area of agreement among the countries was the pandemic’s impact on their organizations’ work, as participants from all countries with at least five responses conveyed that the pandemic had negatively impacted their work (75% in Burkina Faso; 80% in Ghana; 95% in Sierra Leone; and 100% in Senegal, Liberia, The Gambia, and Nigeria). However, the statistically significant association between country and impact on the respondents’ organizations work implies that this impact still varied by country. In both Nigeria and the Gambia, this high impact on respondents’ organizations’ work is correlated with the perception by 100% of respondents in Nigeria and 82% of respondents in The Gambia that alcohol treatment or support is harder to access, suggesting the pandemic’s negative effect on multiple aspects of alcohol harm prevention and treatment. In Burkina Faso, however, while the majority of respondents thought that the pandemic negatively impacted their organizations’ work, only 25% perceived it to be harder to access alcohol treatment/support, therefore, implying a more nuanced overall effect of the pandemic on alcohol harm prevention and treatment. Regardless, these findings underscore the negative impact on the organizations represented in this survey which may have grave implications for an increase in alcohol-related harm in the future. As mentioned, the lack of systems and infrastructure to address alcohol harm in West Africa [5] mean that these organizations involved in alcohol harm prevention take a central role, so a negative impact on their work could translate into an increase in alcohol harm in West Africa, a region already noted with the highest age-standardized alcohol-attributable deaths and disability adjusted life years [14].

Several limitations should be considered when interpreting these findings. First, the participants represented a convenience sample, and no specific response rate can be computed. As such, the results may not be generalizable. Second, the sample was small, preventing any sophisticated statistical analyses. Thus, our findings are not meant to provide precision, but rather a thematic overview of the impact of the COVID-19 pandemic on alcohol use and treatment access as perceived by stakeholders in the region. In addition, participants represented CBOs and NGOs involved in alcohol harm prevention, so these results are based on their perceptions rather than individuals seeking treatment for alcohol misuse or current alcohol drinkers.

Despite these limitations, our findings demonstrate a clear pattern across most of the West African countries. Given the scarce resources to address alcohol harm in the region before the pandemic, the COVID-19 pandemic is further limiting the resources to address alcohol harm in West Africa. The COVID-19 pandemic’s negative impact on alcohol harm prevention and disruption of alcohol support and treatment may translate into an increase in both the short-term and long-term health consequences associated with alcohol in this region. Alcohol is related to a myriad of conditions, so the pandemic could have implications far-reaching into the future in West Africa without strengthening and investing in alcohol harm prevention and alcohol treatment. Ideally, population-level surveillance data are needed to assess drinking levels and alcohol-related harm to conduct time series analyses before and after the pandemic to identify specific needs and intervention targets. However, data on alcohol and related research remain scarce across most of West Africa despite the significant alcohol burden in the region. Another paper using data from this survey proposes topics for a research agenda on alcohol harm prevention in West Africa to serve as a starting point for future research [15] and another paper outlines strategies for assessing the readiness to address alcohol harm [16]. Finally, the findings underscore the importance of connecting with CBOs and NGOs to assess community needs and to strengthen their ability to respond to alcohol harm in vulnerable settings, particularly during the COVID-19 pandemic.

## Conclusion

To our knowledge, this paper is the first study to look at the impact of the COVID-19 pandemic on alcohol use, access to treatment and support services, and alcohol harm prevention in West Africa. Most respondents indicated that they perceived it to be harder for people in their communities to access alcohol treatment or support when needed, and the majority of respondents from West African NGOs and CBOs focused on alcohol harm prevention said that their work was adversely affected because of the pandemic. This finding has important implications for the future landscape of alcohol harm in West Africa, a region with a high burden of alcohol harm and weak systems to prevent alcohol use and harm. This study emphasizes the need to strengthen alcohol harm prevention efforts in West Africa, especially considering the COVID-19 pandemic.

## Data Availability

The datasets analyzed during the current study are available from the corresponding author on reasonable request.

## Abbreviations

CBO: Community-Based Organization
NGO: Non-Governmental Organization
WAAPA: West African Alcohol Policy Alliance
WAAPACAS: West African Alcohol Policy Alliance Capacity Assessment Survey
